# Short Wavelength Cone Opsin Is Not Expressed in the Retina of Arboreal African Pangolin (*Manis tricuspis*)

**DOI:** 10.1155/2016/1535490

**Published:** 2016-05-08

**Authors:** Adejoke J. Adekanmbi, Adefisayo A. Adekanmbi, Oluwole B. Akinola

**Affiliations:** ^1^Department of Anatomy, Faculty of Basic Medical Science, College of Medicine, University of Ibadan, PMB 5017 GPO, Ibadan, Nigeria; ^2^Department of Anatomy, College of Health Sciences, University of Ilorin, PMB 1515, Ilorin, Nigeria; ^3^Department of Neurological Surgery, University College Hospital Ibadan, PMB 5017, Ibadan, Nigeria

## Abstract

This paper reports a study of cone photoreceptors present in the retina of* Manis tricuspis*. Specifically, the LWS (L-) opsin expressed in longwave-sensitive cones and SWS1 (S-) opsin shortwave-sensitive cones were targeted. Vertical sections revealed reactivity to a cone marker, peanut agglutinin (PNA), and to an LWS antibody, but not to an SWS1 antibody. This suggests that the* Manis tricuspis* visual system is not able to discriminate shorter wavelengths from longer wavelengths because the short wavelength cones are not expressed in their retina.

## 1. Introduction

A visual system that can form images is strategic in mammalian survival skills such as searching for food, avoiding predators, and identifying sexual partners [1]. Mammalian rod photoreceptors are used for low light vision while cone photoreceptors are used for daylight and color vision. Color vision in mammals is facilitated by the presence of cone opsins pigments [2]. Placental mammals usually have dichromatic color vision based on the presence of two spectral cone pigment types: the long wavelength sensitive (L-) opsin and the short wavelength sensitive (S-) opsin [3]. In general, primates express three spectral cone pigments giving them excellent color vision [3, 4]. Species like the owl monkey have also been reported to have only one spectral cone opsin pigment [4, 5].

The loss of the S-opsin and/or the L-opsin in different species and its significance have been extensively reviewed [2, 6] and the significance of loss or gain of the photoreceptor opsins is reportedly suited to the different lights required for the survival of the species [2]. Habitat, lifestyle, and genetic incidents are hypothesized to account for these unique losses in nature [2]. Comparing the visual system in different mammals is valuable in its own right and also in understanding conditions considered abnormal in humans that mimic these events in other species and thereby helping us proffer ways to manage or solve these accidents of nature.

Arboreal African pangolins are unique mammals from the homogenous order Pholidota that contains only eight species of the single genus (*Manis*). These animals are generally nocturnal, highly secretive, and endangered or highly threatened [7, 8]. Anecdotal observations have reported a reliance on their sense of smell rather than their visual system to support their insectivorous lifestyle [9, 10]. A study on retinal projections to their brain revealed that they had very tiny optic nerves compared to their brain size and in relation to other common mammals such as rats, monkeys, and cats [9]. Pangolins have very unique keratin scales covering their skin like an epidermal armour and are the only mammals with this adaptation [8]. Confronted with a threat, pangolins curl very tightly into balls [10].

Extending our knowledge on species especially one as endangered as the* Manis tricuspis* may reveal common trends of adaptive specializations under varying lifestyles [11]. In the present work, we used receptor antibodies directed against cone opsin pigments to determine the visual capacity of the arboreal pangolin* Manis tricuspis*.

## 2. Methodology

The present study was carried out on the arboreal African Pangolins. Eight male adult arboreal African pangolins used were captured from the wilds of Olokemeji forest reserve in the rain forest of South-Western Nigeria and their 16 retinas were used in this study. Although pangolins are endangered, there are no restrictions on catching them from the wild, but there are institutional guidelines regulating the use and care of animals for research. Animals were handled in accordance with these institutional ethics on care and use of animals. After euthanasia, the eyes were opened behind the cornea, and the lens and vitreous were removed. The orientation mark was transferred as a dorsal cut in the retina, and the retina was carefully removed from the eyecup. In all eyes, the retina was readily separated from the pigment epithelium without damage to the photoreceptor outer segments.

Sixteen retinas from eight adult male pangolins were fixed in 4% paraformaldehyde (PFA) for 20 minutes and subsequently placed in 0.1 M phosphate buffer (PB, pH 7.4). Cryoprotection was carried out by immersing sections in 30% sucrose for 30 minutes, followed by embedding in Tissue Freezing Medium (Triangle Biomedical). Serial sectioning of embedded retina tissue was carried out in a Leica Cryostat at 20 *μ*m and picked up onto slides.

Serial sections were incubated successively in blocking solution (3% normal donkey serum and 0.3% Triton-X-100 in phosphate buffered saline [PBS]) for 1 hour at room temperature, followed by incubation in primary antibodies diluted in PBS containing 3% normal donkey serum and 0.3% Triton-X-100 for 12–14 hours at 4°C. Tissue sections were washed in PBS twice.

SWS1 opsin was detected with the goat antiserum sc-14363 which had been raised against a 20 aa N-terminal epitope of the human S (blue) cones at dilution of 1 : 500 opsin (Santa Cruz Biotechnology, Inc., Santa Cruz, CA, USA). The LWS opsin was detected with the rabbit antiserum JH 492 raised against a C-terminal epitope of the human cone opsin (kindly provided by J. Nathans, John Hopkins University School of Medicine, Baltimore, to the Sanes Lab, Harvard University, MA, USA) at dilution of 1 : 1,000 and a cone marker peanut agglutinin, PNA (biotinylated PNA at dilution of 1 : 500, Sigma, USA). All antibodies were aliquots previously stored at −20°C before use.

Sections were subsequently transferred into fluorophore-labeled secondary antibodies (Alexa 488, Alexa 564, and Alexa 594, Molecular Probes, Eugene, OR, USA) for 1 to 2 hours at room temperature. Sections were coverslipped. Optical and fluorescent retinal images were collected using an Olympus Fluo View 1000 laser scanning confocal microscope with a 40x objective without oil immersion (Olympus America Incorporated, NY, USA). Images of sections were acquired as a Z-stack by collecting light from thin (1 *μ*m apart) regions of interest. Images were acquired with contrast and brightness settings appropriate for filling the 8-bit coding image range recognizable by the software FIJI. The zoom of the acquired image was in a ratio of 1 : 1 and florescence images were acquired at 1024 × 1024 (pixels) saved as OIF files in order to make images recognizable by FIJI on image J (NIH, USA).

## 3. Results

**Figure 1:**
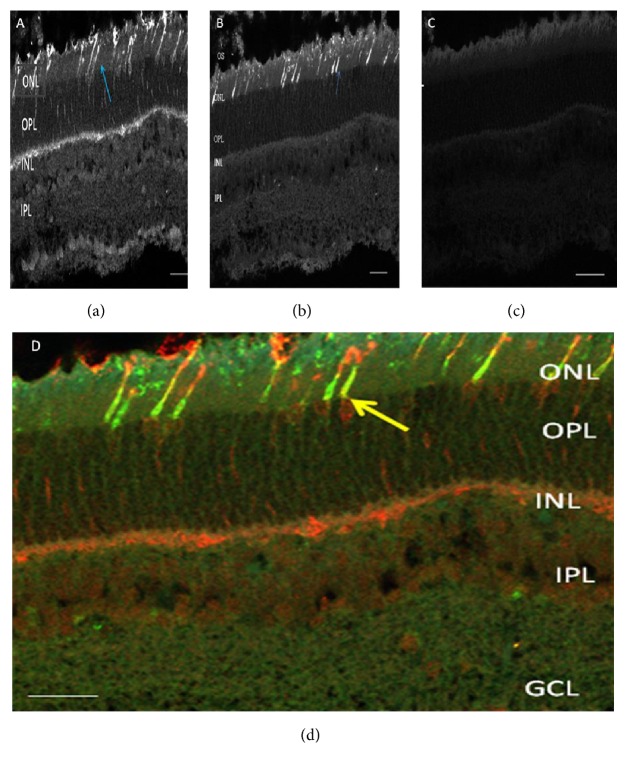
(a) Strong immunoreactivity with PNA cone marker in ONL and cone terminals across all vertical sections of pangolin retina. (b) Strong expression of L-cone opsin across all vertical sections of pangolin retina. (c) S-cone opsin absent across all vertical sections of pangolin retina. Blue arrow indicates cone photoreceptors, PNA is peanut agglutinin, INL is inner nuclear layer, OPL is outer plexiform layer, ONL is outer nuclear layer, L— is long wavelength, and S- is short wavelength. Scale bar 20 *μ*m. (d) Triple labeling showed strong immunoreactivity with PNA cone marker (orange) in the ONL and in the terminals in the INL, across all vertical sections of pangolin retina. L-cones opsins are strongly expressed in the ONL (green). Short wavelength cones are not expressed across all vertical sections of the retina. Yellow arrow indicates cone photoreceptor. D GCL iOPL is outer plexiform layer, IPL is inner plexiform layer, ONL is outer nuclear layer, and INL is inner nuclear layer. Scale bar is 20 *μ*m.

Serial sectioning and immunostaining of retinal tissue of the* Manis tricuspis* showed strong immunoreactivity with PNA, a cone opsin marker in the outer nuclear layer and in the terminals of the cones in the inner nuclear layer (Figure 1(a)). Antibody to long wavelength cone opsin showed strong immunoreactivity with cones in the outer nuclear layer but not in the terminals (Figure 1(b)), while antibody to short wavelength cone opsin did not immunolabel any cone in the outer nuclear layer nor in the terminals either as a constant fraction or as a gradient across the retina even though the marker used is a robust antibody (Figure 1(c)).

In triple labeling experiments, the PNA labeled cones were the same population labeled with L-cone marker hence accounting for ~100% of all cones without any cone expressing the S-cone opsin across all retinal sections (Figure 1(d)).

## 4. Discussion

The importance of an adequate color vision capacity is emphasized by its persistence throughout the long history of mammalian species especially because it plays a useful role in supporting survival by giving them keen discriminatory abilities [12]. The absence of short wavelength cones in* Manis tricuspis* is likely adequate for their visual information processing. Cones are the photoreceptors that give the capacity for color vision and specifically color discrimination requires the presence of two or more types of photoreceptor with spectrally discrete visual pigments [13]. Cones are categorized into spectral types containing different visual pigments and they send spectral information through interneurons to appropriate ganglion cells [14].

Generally, vertebrates have four types of cone visual pigment located in four spectral cone types: SWS1 (the short wavelength sensitive 1 which is in the range of near-ultraviolet to violet), SWS2 (the short wavelength sensitive 2, which is in the range of violet to blue), RH2 (the middle wavelength sensitive, which is in the range of the green), and LWS (the long wavelength sensitive, which is in the range of yellow to red) [12, 13]. A visual pigment consists of a protein, the opsin which surrounds a chromophore, and the spectral sensitivity of a given pigment is determined by the amino acid sequence of the opsin [2].

Many diurnal bonefishes, reptiles, and birds possess all four cone types and thus the potential for tetrachromatic color vision. Some mammals have lost the opsin classes RH2 and SWS2; instead, they only retained the classes LWS and SWS1; others like monotremes have SWS1 and retained the SWS2 [2, 14]. Consequently, the most common mammalian condition is dichromatic color vision, on the basis of L-cones and S-cones, therefore allowing discrimination of shorter wavelengths from longer wavelengths but no discrimination between longer wavelengths [5]. The implication for the* Manis tricuspis* is total loss of color vision, with rods serving scotopic and L-cones serving photopic vision. Overall visual acuity is not necessarily affected as S-cones in other retinas like in primates form only a small fraction of the total cone compliment [15]. This may be why S-cones, not L-cones, are generally lost. A number of eutherian mammals have shifted their peak sensitivity SWS1 from near-ultraviolet to violet or blue, that is, to the position of the lost SWS2 pigment, and the LWS pigment sensitivity ranges from green to red depending on species [10], suggesting that spectral tuning, effectuated by amino acid changes in the opsins, is under strong selective pressure. In majority of marsupials, they have retained a UV-sensitive SWS1 pigment and in old world primates including man, trichromatic color vision reevolved by a duplication of the LWS opsin gene to yield two spectrally discrete LWS opsins, the green and red cone opsins [10].

Previous study on the* Manis tetradactyla* retina reported that they have evolved towards secondary monochromacy [11]. The* Manis tricuspis* and* Manis tetradactyla* both belong to the order Pholidota and this may very well be a unique characteristic of the species in that order. Lack of expression of S-cone opsin in the* Manis tricuspis* limits its color vision capacity; specifically, the ability to reliably distinguish different levels of spectral energies reaching the eye for higher center analyses and interpretation of the visual environment requires the presence of short wavelength and long wavelength cones [12, 13]. This implies that the absence of S-cones in the* Manis tricuspis* hampers this basic requirement for color discrimination and gives them the status of a monochromatic mammal. The presence of only the L-cones has been documented in some other species too [2]. S-cones are present in representative species from all other vertebrate classes except cartilaginous fishes [15]; their loss is reportedly common in most aquatic mammals and some nocturnal terrestrial species [16]. The peak spectral sensitivity of S-cones depends on the spectral characteristics of the pigment present; for example, in the ultraviolet (UV) sensitive, it peaks around 360 nm, while in the violet region of the spectrum, it is greater than 380 nm and the shift between species is probably the result of vertebrate evolution. In all cases, the shift was generated by just one or a few replacements in tuning-relevant residues [16]. Although photopigments are highly conserved across vertebrates, striking variations appear, disappear, and emerge again in altered form in the process of evolution [12]. These occurrences or changes that may or may not come along with other alterations in the visual system often lead to profound variations in the nature and mechanism of color vision processing among vertebrates [12].

Over the years, the use of antibodies targeting the cone opsins has become quite popular and effective; these are directed against conserved epitopes and they recognize the respective opsin types across different species. Immunocytochemical labeling of the cone opsins has two major advantages. First, it allows a determination of the population properties of cones, for example, L- and S-cones, including their topographic distribution across the retina (Figures 1(a), 1(b), 1(c), and 1(d)). These properties have important functional consequences, and they cannot be obtained by other methods. Secondly, the antibodies work in fixed tissue; hence, the presence and distribution of the cone types can be assessed in species that are not easily available for physiological or behavioral experiments. Studies using opsin antibodies have greatly increased our knowledge about the diversity of cone arrangements across mammals. They have identified a number of species with particularly interesting cone properties, which have subsequently been scrutinized by molecular and physiological approaches. The first evidence that there could be some nonhuman species similar to human tritanopes (humans that lack functional S-cones) came from an opsin antibody labeling study of two species of nocturnal primates: the platyrrhine owl monkey (*Aotus trivirgatus*) and the strepsirrhine bushbaby (*Galago garnettii*) [16]. A subsequent study of the* Aotus* monkey, involving both behavioral and electroretinogram (ERG) measurements, similarly failed to detect any functional evidence for the presence of S-cones. Hence, it has been hypothesized that pigment arrangement in this monkey might be analogous to that of human tritanopes since the SWS1 opsin genes in this species have defects that disturb the production of functional S-cone pigments [5]. This suggestion was made when the analysis of the sequences of the SWS1 opsin genes in both owl monkeys and bushbabies revealed that these genes (a) were highly homologous to the human S-cone opsin gene but (b) harbored mutational changes that would make the production of fully functional photopigments impossible [13]. The nature of the mutational changes was different in the two species but both featured the presence of nucleotide insertions and deletions that introduced premature stop codons [13]. Over the past 20 years, several mammalian species have been reported to have lost S-cones based on studies using techniques such as gene sequences, functional measures like ERG recordings, and opsin antibody labeling of photoreceptors or from observations obtained from direct behavioral tests of vision [10]. To date, evidence for mammalian species from five orders that have lost S-cone function as a result of opsin gene mutations has been reported [11, 15]. For example, the SWS1 gene is reported to be inactivated by mutational changes in the monotremes platypus* Ornithorhynchus anatinus* and echidna* Tachyglossus aculeatus*[4, 15]. Other examples include rodents, whales, carnivores, and primates [15, 17].

A limitation of this study is the inability to complement our result with a molecular approach to establish lack of SWS1 opsin transcript or the presence of an SWS1 pseudogene in the genome and functional measures like ERG recordings due to insufficient funding. A future direction for this study is to make observations from direct behavioral tests of color vision in the* Manis tricuspis*.
